# DL-3-n-butylphthalide delays the onset and progression of diabetic cataract by inhibiting oxidative stress in rat diabetic model

**DOI:** 10.1038/srep19396

**Published:** 2016-01-13

**Authors:** Fuxu Wang, Jia Ma, Fei Han, Xiujin Guo, Li Meng, Yufeng Sun, Cheng Jin, Huijun Duan, Hang Li, Ying Peng

**Affiliations:** 1 Department of Hematology, the Second Hospital of Hebei Medical University, 215 Western Heping Road, Shijiazhuang 050000, China; 2 Department of Ophthalmology, the Second Hospital of Hebei Medical University, 215 Western Heping Road, Shijiazhuang 050000, China; 3 Department of Digestology, the Second Hospital of Hebei Medical University, 215 Western Heping Road, Shijiazhuang 050000, China; 4 Laboratorical center for Electron Microscopy, Hebei Medical University, 361 Eastern Zhongshan Road, Shijiazhuang 050017, China; 5 Department of Histology and Embryology, Hebei Medical University, 361 Eastern Zhongshan Road, Shijiazhuang 050017, China; 6 Department of Pathology, Hebei Medical University, 361 Eastern Zhongshan Road, Shijiazhuang 050017, China; 7 State Key Laboratory of Bioactive Substances and Functions of Natural Medicines, Institute of Materia Medica, Chinese Academy of Medical Sciences & Peking Union Medical College, Beijing 100050, China

## Abstract

DL-3-n-butylphthalide (NBP) is a therapeutic drug used for ischemic stroke treatment. Here, we investigated the impact of NBP on the development of rat diabetic cataract induced by intraperitoneal injection of streptozotocin (STZ). NBP was then administrated by oral gavage for nine weeks. Cataract development was monitored through ophthalmoscope inspections. The levels of blood glucose and serum reactive oxygen species (ROS), malondialdehyde (MDA) and 8-Hydroxydeovexyguanosine (8-OHdG) were measured. Total and soluble protein and oxidative stress parameters, such as 2, 4- dinitrophenylhydrazone (DNP), 4-hydroxynonenal (4-HNE) and MDA in the lenses were determined by Western blot and thiobarbituric acid analyses. The expressions of NF-E2-related factor 2 (Nrf2) and its downstream antioxidant enzymes, thioredoxin (TRX), Catalase and nuclear accumulation of Nrf2 were determined by Western blot and immunohistochemistry analyses. We showed that NBP treatment significantly improved the cataract scores, the levels of DNP, 4-HNE, and MDA in the lens compared to the non-treated groups. NBP also enhanced the expressions of Nrf2, TRX and catalase in the lens of diabetic rats. In addition, NBP treatment also decreased levels of blood glucose, serum MDA and 8-OHdG. These results suggested that NBP treatment significantly delayed the onset and progression of diabetic cataract by inhibiting the oxidative stresses.

Cataract is characterized by cloudiness and opacification of the eye’s natural lens. It is the leading cause of blindness in the world^1^. Diabetes mellitus is a major risk factor for cataract^2^. A great deal of studies have demonstrated that chronic hyperglycemia-induced overproduction of reactive oxygen species (ROS) played a central role in the pathogenesis of diabetic complications including diabetic cataract^3^,^4^,^5^. High level of ROS directly disturbs physiological functions of cellular macromolecules, and subsequently leads to lens opacification.

Currently, surgery of cataract removal and intraocular lens implant is the main treatment for diabetic cataract. However, surgery may lead to a lot of serious postoperative complications such as infection, corneal edema and increased intraocular pressure, especially in the elderly people and in hyperglycemia conditions^6^. Therefore, it is necessary to develop effective therapeutic strategies for the prevention and treatment of diabetic cataract.

ROS damages inside cells are eliminated by endogenous antioxidant enzymes that are regulated by the antioxidant responsive element (ARE), a cis-acting element within the regulatory region of antioxidant and phase II detoxicant genes^7^. Notably, NF-E2-related factor 2 (Nrf2) is an activator of ARE. Thus, Nrf2 is one of the most important transcription factors that stimulate endogenous antioxidant against excessive ROS^8^. Some studies indicated that activation of Nrf2 and its downstream antioxidants enzymes mitigated ROS damages in diabetic nephrology^9^, diabetic neuropathy^10^ and diabetic atherosclerosis^11^. However, the relationship between Nrf2 and diabetic cataract is not clear.

NBP is a widely used clinically therapeutic drug for ischemic stroke. It is a multiple-target neuroprotective agent that significantly reduces oxidative damages, improves mitochondrial function, decreases neuronal apoptosis and inhibits inflammation^12^,^13^. We have previously showed that NBP upregulated the expressions of Nrf2 and its downstream antioxidants enzyme, heme oxygenase-1 (HO-1) in a mouse model of amyotrophic lateral sclerosis^14^. In the present study, we hypothesized that NBP could improve hyperglycemia-induced diabetic cataract by increasing the expressions of Nrf2 and its downstream antioxidants. We therefore evaluated the effect of NBP on the development and progression of hyperglycemia-induced diabetic cataract and the possible mechanisms involved in these processes using STZ-induced diabetic rats.

## Results

### NBP increased body weight and reduced the blood glucose level in diabetic rats

**Figure 1:**
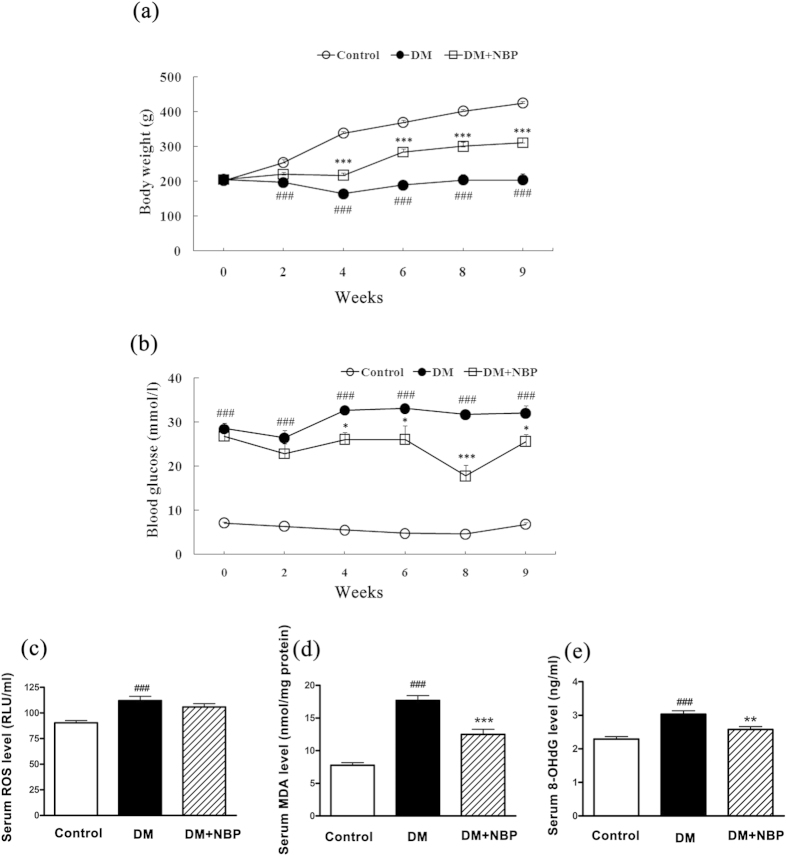
Blood and serum biochemical parameters of different groups at different time points (**a**) The average body weight. **(b)** The average blood glucose level. **(c)** Serum ROS levels at nine weeks post-diabetes. **(d)** Serum MDA levels at nine weeks post-diabetes. **(e)** Serum 8-OHdG levels at nine weeks post-diabetes. Control: normal rats (n = 15); DM: diabetic rats (n = 13–15); DM + NBP: diabetic rats with NBP treatment (n = 12–15). Data are expressed as mean ± SEM. ^###^P < 0.001 vs. Control; *P < 0.05 vs. DM; **P < 0.01 vs. DM, ***P < 0.001 vs. DM.

No rats in the control group died and no statistical mortality was observed in the DM (2 of 15) and DM + NBP (3 of 15) groups during the experiment. The body weight of the diabetic groups was decreased significantly from the 2^nd^ week to the 9^th^ week compared to the control group (p < 0.001). Interestingly, the reduction of body weight was remarkably ameliorated by NBP treatment from the 4^th^ week to the 9^th^ week (p < 0.001 Fig. 1a). The blood glucose levels of both diabetic groups were significantly higher than the control group (p < 0.001). After the administration of NBP, the glucose levels were markedly reduced compared to non-NBP treated DM group (4^th^ week: p < 0.05; 6^th^ week: p < 0.05; 8^th^ week: p < 0.001; 9^th^ week: p < 0.05, respectively Fig. 1b).

### NBP ameliorated the serum oxidative stress in the diabetic rats

The biomarkers of oxidative damages to lipids and DNA were detected by measuring the levels of ROS, MDA and 8-OHdG in the serum at the end of nine weeks. The results showed that serum levels of ROS (Fig. 1c); MDA (Fig. 1d) and 8-OHdG (Fig. 1e) in diabetic groups were obviously elevated compared to the control group. Following treatment with NBP, the concentrations of MDA and 8-OHdG were significantly reduced compared to that of non-treated diabetic rats at nine weeks (p < 0.001; p < 0.01, respectively). Though the difference did not reach to the significance, the rise in ROS level was also ameliorated in the NBP-treated DM group.

### NBP alleviated the formation and progression of cataract in diabetic rats

**Figure 2:**
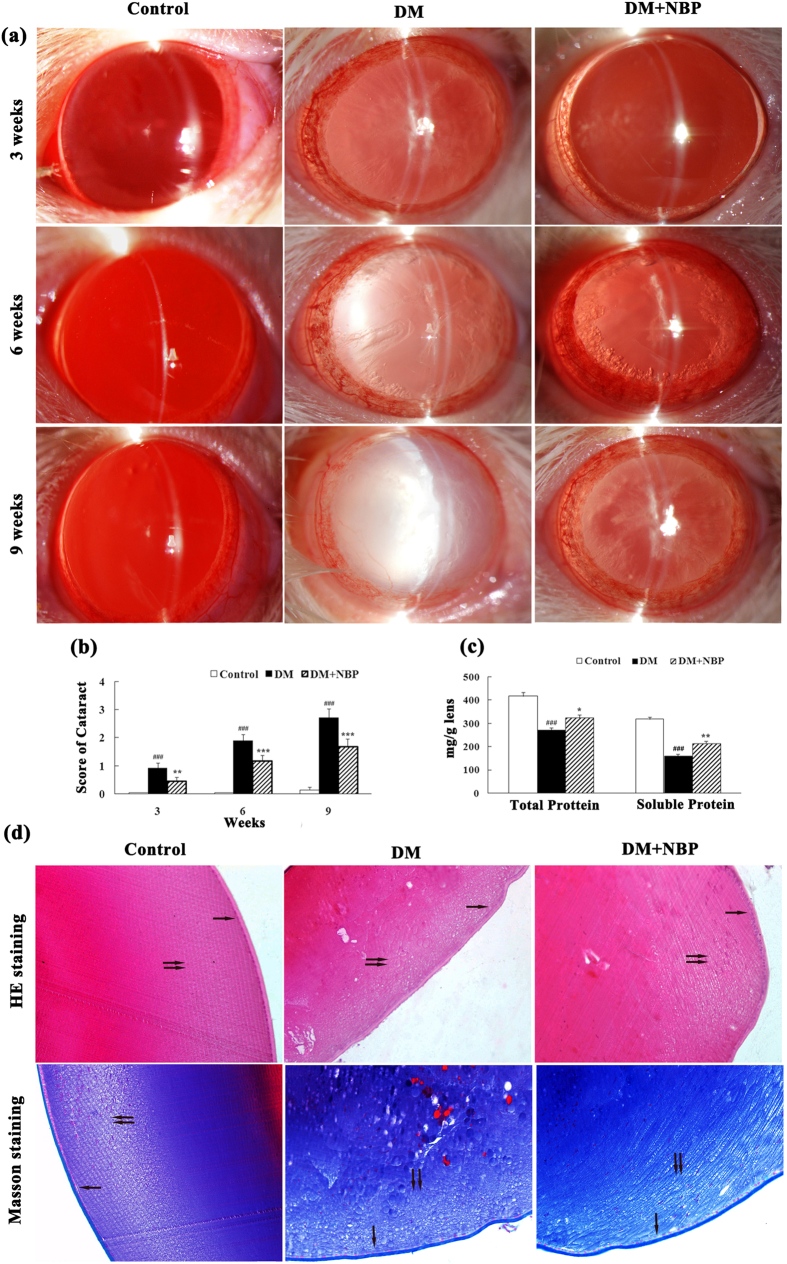
Formation and progression of cataract in different groups at different time point (**a**) Representative photographs of lenses at different time points. (Control, n = 15; DM, n = 13–15; DM + NBP, n = 12–15). (**b**) Average scores of cataract (Control, n = 15; DM, n = 13; DM + NBP, n = 12). (**c**) Lens total and soluble proteins of each group at nine weeks post-diabetes (n = 6 for each groups) (**d**) Representative histological photographs of lenses at nine weeks post-diabetes (n = 6 for each groups). The lenses tissue sections were stained with H&E and Masson and examined under a light microscope. One black arrow: lens epithelial cells; two black arrows: lens cortical region (magnification x200). Control: normal rats; DM: diabetic rats; DM + NBP: diabetic rats with NBP treatment. Data are expressed as mean ± SEM. ^###^P < 0.001 vs. Control; *P < 0.05 vs. DM; **P < 0.01 vs. DM; ***P < 0.001 vs. DM.

The onset of cataract was observed after three weeks of STZ injection by slit lamp examination and progressed to mature cataract by 9^th^ week in some diabetic animals. Since lenses were in different stages of cataract formation in a given group at a given time, we have averaged the stages at the given time (3^rd^ week; 6^th^ week and 9^th^ week) in order to observe the onset and progression of cataract in all the groups (Fig. 2a). Interestingly, a significant decrease in the average score of cataract was detected in NBP-treated DM group compared to non-treated DM group (p < 0.01 at 3^rd^ week; p < 0.001 at 6^th^ and 9^th^ week, Fig. 2b). All the lenses in the control group appeared to be clear and normal during the experimental period.

Alteration of protein profile and insolubiliztion of total protein have been considered to be the ultimate changes that result in lens opacification. We therefore measured the total and soluble protein contents in the lens at the end of nine weeks following treatment. In the DM group, there was a significant decrease in the total and soluble protein levels compared to the control group. However, treatment with NBP remarkably up-regulated the total and soluble protein levels of diabetic lens (total p < 0.05; soluble p < 0.01. Fig. 2c).

The results from H&E staining (Fig. 2d) and Masson staining (Fig. 2d) were very similar in the lens sections. We chose the Masson staining for further analysis because it was brighter and clearer when examining the pathological changes. In the control group, the lens epithelial cells, fiber cells and cortical architecture were orderly arranged. However, in the diabetic group, the epithelial and cortical fiber cells were swelling and disorderly arranged, the number of the cell nuclei was remarkably decreased and a large number of distinctive vacuolar changes in the cortical region were detected. In the NBP-treated DM group, epithelial cell swelling was attenuated, and fewer vacuoles were observed in the cortical region. The pathological changes in lens fiber were effectively prevented, and the decrease in the number of cellular nuclei was also alleviated compared to the non-treated DM group.

### NBP mitigated the damage of oxidative stress in the lenses of diabetic rats

**Figure 3:**
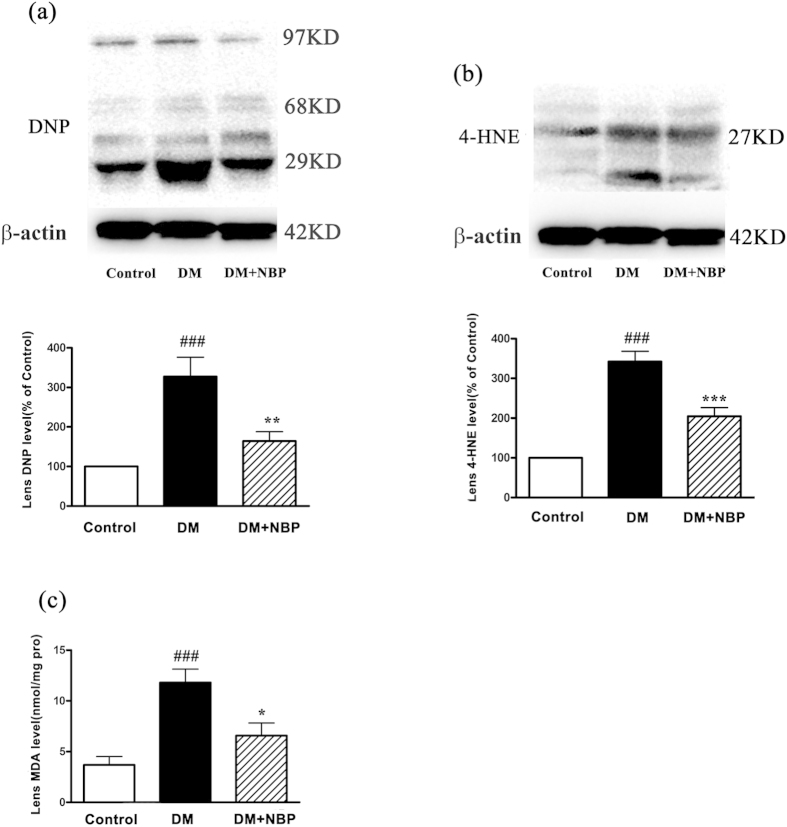
Oxidative stress parameters of the rat lenses at nine weeks post-diabetes (**a**) Relative levels of lenses DNP to β-actin by Western blot assays and quantitative analysis This is a cropped blot and full-length blots are presented in Supplementary information Figure 1. (**b**) Relative levels of lenses 4-HNE to β-actin by Western blot assays and quantitative analysis. This is a cropped blot and full-length blots are presented in Supplementary information Figure 2. (**c**) MDA levels of lenses at nine weeks post-diabetes. Control: normal rats; DM: diabetic rats; DM + NBP: diabetic rats with NBP treatment. Data shown are representative images or mean ± SEM of each group (n = 6). ^###^P < 0.001 vs. Control; *P < 0.05 vs. DM; **P < 0.01 vs. DM; ***P < 0.001 vs. DM.

The oxidation status of lens proteins was detected by carbonyl reaction products 2, 4-dinitrophenylhydrazone (DNP) and the lipid peroxidation of lenses was detected by the production of lipid peroxide 4-hydroxynonenal (4-HNE) and MDA. As shown in Fig. 3a, the DNP level was increased by 228% in the DM group compared to the control group (p < 0.001); however, feeding the rats with NBP decreased the DNP level by 50% compared to the DM group (p < 0.01). In addition, a robust increase of 4-HNE levels in the lens was observed in DM rats by 242% compared with the control animals (p < 0.001); and NBP treatment significantly attenuated the elevation of 4-HNE levels in diabetic rats by 40% (p < 0.001, Fig. 3b). The lens MDA levels in DM group were increased over two fold compared to the control group (p < 0.001); while the MDA level was dramatically decreased by 45% after NBP treatment (p < 0.05 Fig. 3c).

### NBP up-regulated the expression of Nrf2 and downstream antioxidants TRX and Catalase in the lens of diabetic rat

**Figure 4:**
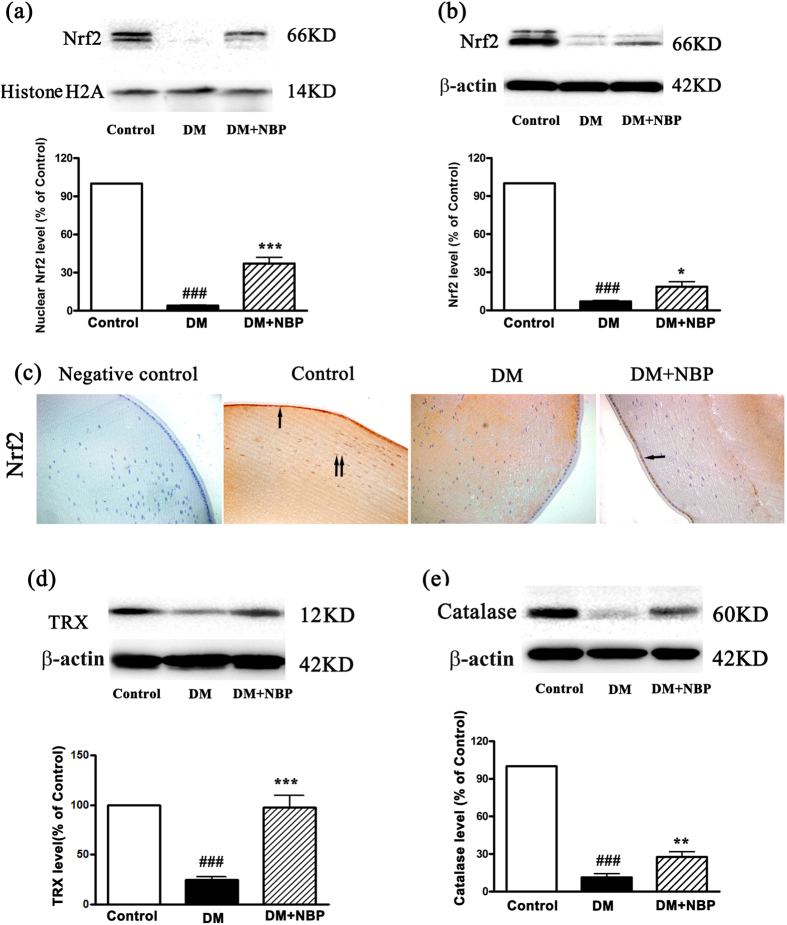
Expression of Nrf2,TRX and Catalase in the lens of rats at nine weeks post-diabetes. **(a)** Relative levels of nuclear Nrf2 to histone-H2A by Western blot and quantitative analyses. This is a cropped blot and full-length blots are presented in Supplementary information Figure 3. (**b**) Relative levels of Nrf2 to β-actin by Western blot assays and quantitative analysis. This is a cropped blot and full-length blots are presented in Supplementary information Figure 4. (**c**) The expression of Nrf2 in the lens by immunochemistry. One black arrow: Positive Nrf2 staining in lens epithelial cells; two black arrows: Positive Nrf2 staining in fiber cells (magnification x200). **(d)** Relative levels of TRX to β-actin by Western blot assays and quantitative analysis. This is a cropped blot and full-length blots are presented in Supplementary information Figure 5. **(e)** Relative levels of Catalase to β-actin by Western blot assays and quantitative analysis. This is a cropped blot and full-length blots are presented in Supplementary information Figure 6. Control: normal rats; DM: diabetic rats; DM + NBP: diabetic rats with NBP treatment. Data shown are representative images or mean ± SEM of each group (n = 6). ^###^P < 0.001 vs. Control; ^*^P < 0.05 vs. DM; ^**^P < 0.01 vs. DM; 3^***^P < 0.001 vs. DM.

To explore the antioxidant mechanism of NBP on the diabetic cataract, the expressions of Nrf2 and its downstream antioxidant enzyme were detected. The expressions of nuclear and cytoplasmic Nrf2 was hardly detected in the diabetic lens compared to the control group by Western blot; however, a higher level of Nrf2 expression in both nucleus (by 10 fold) and in cytoplasm (by 3 fold) were detected in the lens of NBP-treated DM group (Fig. 4a,b). To further test the expression of Nrf2 localization, we performed immunohistochemistry. As demonstrated in Fig. 4c, Nrf2 was mainly expressed in the nucleus and cytoplasm of the epithelial and fiber cells in the control lenses; while in the lenses of diabetic rats little expression of Nrf2 was detected in the epithelial and fiber cells. Remarkably, the increased Nrf2 expression was observed in the NBP-treated DM group, especially in lens epithelial cells.

As Nrf2 is a transcription factor of the antioxidants, we tested its downstream antioxidant proteins: TRX, Catalase, HO-1, γ-glutamylcysteine synthethase (γ-GCS) and NAD (P) H: quinone oxidoreductase 1 (NQO1). No statistical relationship of Nrf2 with HO-1, γ-GCS and NQO1 expressions were found in the lens of these three groups (data not shown). However, in concordance with Nrf2, the expression of TRX and Catalase were significantly decreased in the DM group while remarkably increased 4 folds (TRX, Fig. 4d) and 2.5 folds (Catalase, Fig. 4e) respectively following NBP treatment.

## Discussion

In this study, we demonstrated that NBP administration effectively inhibited the development and progress of STZ-induced diabetic cataract by morphological observations, histological examinations, and biochemical analyses. We also found that NBP reduced the blood glucose levels in the diabetic rats. Although the exact mechanisms of NBP involved in reducing glucose levels and preventing diabetic cataract are not yet clear, we assumed that the decreased serum levels of oxidative stress parameters, such as ROS, lipid peroxidation product MDA, DNA oxidative derivative 8-OH-dG might play the critical roles in these processes.

The mechanism of lens damages in the diabetes is complex and has been the subject of much debate. A previous study has found that NBP improved cognitive function by up-regulating the expression of NR2B in STZ-induced diabetic rats^15^. Several studies also showed that diabetic patients, even with well-controlled blood glucose levels, are still sensitized to cataract formation compared to non-diabetics^16^,^17^. Furthermore, despite well-controlled blood glucose levels, diabetic complications still inevitably take place via several mechanisms including excessive generation of free radicals in patients who suffer from diabetes mellitus^18^. In our study, we found that although the blood glucose level of diabetic rats decreased when treated with NBP, it is still higher than the threshold (16.7 mmol/L) that is necessary for the cataract development^19^. On the other hand, we found that the cataract score did not correspond with glucose levels in diabetic rats whether they were treated with NBP or not. In some diabetic rats, we also observed that the development of cataract was not same in both eyes of one rat although the blood glucose level was same. Interestingly, NBP administration significantly decreased the cataract scores. Therefore, the delay in onset and the inhibition of progression of cataract after the administration of NBP in diabetic rats are possibly due to other factors in addition to its glucose lowering property.

ROS damages proteins, lipids, polysaccharides and nucleic acids within ocular tissues that are all associated with cataract formation^20^. Therefore, prevention of oxidative stress damage by antioxidants is considered a viable means of medically offsetting the onset and progression of this vision-impairing disease^21^. In fact, administrations of various nutritional and metabolic antioxidants, such as curcumin^22^, pyruvate^23^, caffeine^24^, and glycine^25^ have been demonstrated to prevent cataract formation in many experimental animal cataract models. However, owing to lack of success in patients, no drug has yet been approved for clinical use. In the present study, we examined three representative oxidative stress biomarkers, DNP (protein peroxidated production), MDA and 4-HNE (lipid peroxidated production) to evaluate the lens oxidative stress damage level. We found that the oxidative stress damage accumulation in the diabetic lens could be reduced with NBP treatment. These results revealed for the first time that NBP, a widely used therapeutic drug for ischemic stroke in China might effectively inhibit the development of STZ-induced diabetic cataract by protecting against oxidative stress and therefore highlight a promising therapeutic use of NBP to prevent or treat cataract.

Previous studies indicated that disruption of the balance between ROS production and scavenging leads to cellular apoptosis, which is associated with cataract formation^26^. Therefore, cellular defenses have been suggested as important factors in protecting the lens cells against oxidative stress and postponing cataract formation. One of the most important cellular defense mechanisms against excessive ROS is regulated by Nrf2, a transcription factor that regulates many of the antioxidant defense genes, including TRX, HO-1, Catalase, NQO1, γ-GCS and other antioxidant enzymes. Under physiological conditions and low oxidative stress environment, Nrf2 is localized in the cytoplasm with the suppressor protein Kelch-like ECH-associated protein (Keap1), and it is degraded by the ubiquitin proteasome pathways. The oxidative and electrophilic stresses alter the Nrf2-Keap1 complex and promote the translocation of Nrf2 into the nucleus. In the nucleus, Nrf2 binds to the ARE to activate the multiple antioxidant gene expressions^8^. Thus, maintaining its level and activities are considered useful in protecting the tissues against oxidative stress and the consequent onset of pathogenetic pathways. Elevated Nrf2 expression was observed in both cytoplasm and nucleus in the glomeruli of diabetic rats and mesangial cells cultured in high glucose levels in our previous studies^27^,^28^. Similar patterns of Nrf2 expression under high glucose and oxidative stress circumstances were observed in many other studies such as the glomeruli of human diabetic nephropathy patients^29^, renal proximal tubule cells under high glucose level *in vitro*^30^, the brain tissue of type 1 diabetic rats^31^ and the aorta of genetic type 1 diabetic OVE26 mouse model^32^. However, in this study, we found that Nrf2 was hardly detected in cytoplasm and nucleus in the diabetic lens when compared to normal controls. We assumed that the expression of Nrf2 might be varied in different tissues and different physiologic consequences are likely related to diverse transcriptional pathways among different tissues^8^,^33^. In addition, ROS production in the lens was higher compared to any other parts of the body, thus, the lens becomes more susceptible to oxidation and less able to repair oxidative damages^34^. The super-production of ROS might exceed the Nrf2 dependent antioxidant defense protection system, altered the redox-balance towards lens oxidation and resulted in the oxidation of the lenses and cataract formation. Palsamy *et al.*^35^ found a significant high level of demethylated DNA in the Keap1 promoter in the cataractous lenses from diabetic patients. They presumed that the demethylation of the CpG islands in the Keap1 promoter might activate the expression of Keap1 protein, which then decreased Nrf2 activity by increasing Nrf2 degradation. NBP has been shown to up-regulate Nrf2 transcription and consequently exerts an antioxidant effect in a mouse model of amyotrophic lateral sclerosis^14^. In this study, we found that NBP up-regulated the Nrf2 expression in the lens cytoplasm and the nucleus. We also found the relative levels of Nrf2, TRX and Catalase were significantly elevated in the lens of NBP treated diabetic rats. Apparently, NBP activated the Nrf2-ARE pathway and promoted the downstream antioxidants, TRX and Catalase expressions in the lens of diabetic rats. Given that TRX and Catalase are strong antioxidant enzymes, higher expressions of them may in turn reduce the hyperglycemia-related lens oxidative stress injury in diabetic rats. Indeed, a previous study has found that cataractous lenses lost more than 70% TRX activity compared with control^36^ and Catalase protected human lens epithelial cells against H2O2-induced oxidative stress^37^. Therefore, activating the Nrf2-ARE pathway may be a valuable therapeutic target for effective intervention in diabetic cataract formation.

In summary, our data indicated that NBP administration reduced the hyperglycemia- related oxidative stress damage, which was associated with enhanced expressions of Nrf2, and its downstream antioxidants TRX and catalase in the lens of diabetic rats. Therefore, NBP shows promising preclinical potential as a pro-antioxidant for diabetic cataract.

## Research design and Methods

### Induction of diabetes and drug treatment

Male Sprague-Dawley (SD) rats of six weeks old (200 ± 15 g) were purchased from Hebei Laboratory Animal Corp. Ltd (Shijiazhuang, China, Certificate No.137026). Rats were housed in a specific pathogen free facility. All the experiments were carried out in compliance with the Regulations of Experimental Animal Administrations issued by the State Committee of Science and Technology of the People’s Republic of China. Rats received a single dose of streptozotocin (STZ, Sigma, USA, 65 mg/kg, freshly prepared in 0.1 M citrate buffer, pH 4.5) by intraperitoneal injection. For the control, the rats received the injection with sodium citrate buffer only according to our previous study. Individual animal with blood glucose concentrations ≥16.7 mol/L after 72 hours of STZ-injection were considered diabetic and used for this study.

NBP was obtained from CSPC NBP Pharmaceutical C., LTD and was dissolved in vegetable oil and administered to the diabetic rats in three dosages (40 mg/kg; 80 mg/kg; 160 mg/kg) according to the data from our preliminary experiment. We found that 40 mg/kg of NBP has no effect on oxidative stress inhibition and cataract alleviation in diabetic rats; while 160 mg/kg of NBP induced higher mortality compared to diabetic controls (data not shown). 80 mg/kg NBP showed a better vitality, body weight and lower blood glucose level. Interestingly, these parameters were not influenced in non-diabetic normal rats (data not shown). Therefore, 80 mg/kg of NBP was chosen in this study. Diabetic rats were randomized and divided into two groups: one received NBP treatment (DM + NBP group, n = 15); and the other received vegetable oil alone (DM group, n = 15). NBP was administered by oral gavage 5 d/week at a dose of 80 mg/kg body weight. Control and DM groups received oral gavage in the same manner with vegetable oil only. Fasting blood glucose level and the body weight of each rat were measured every two weeks.

### Slit lamp examination and cataract grading

Eyes were examined every week using a slit lamp biomicroscope (TOPCON SL-D7 slit lamp, Japan) on dilated pupils and scored every three weeks. We only chose some representative lens to get photos by slit lamp at different time points. Those animals that were chosen to take photos were anesthetized because the examination would take some times. Initiation and progression of lenticular opacity was graded according to the classification of lens opacification as followings^38^: clear normal lens (Grade 0); peripheral vesicles (Grade 1); peripheral vesicles and cortical opacities (Grade 2); diffuse central opacities (Grade 3); and mature cataract (Grade 4).

### Sample collections and processing

After nine weeks treatment, animals were sacrificed and the eyeballs were removed for biochemical analysis. The lens of the left eye was fixed in 4% paraformaldehyde in 0.01 mol/L PBS for histological and immunohistochemistry examination. The lens from the right eye was stored at −70 °C for subsequent analysis. A 10% homogenate was prepared from half of the lens in 50 mM phosphate buffer (pH7.4). The activity of the lens enzymes and soluble protein were measured in the soluble fraction of the lens homogenate (12,000 × g at 4 °C, 20 min) while the lens MDA and the total protein were determined in the total homogenate. Nuclear proteins were extracted from the other half of the lens using the nuclear and cytoplasm extraction reagents (Thermo, USA).

Blood samples were collected from the femoral vein after removal of the eyeballs. Serum was extracted from EDTA-treated whole blood samples.

### Biochemical measurements

Serum ROS levels were measured using luminol chemiluminescence detection kit (GENMED SCIENTIFICS, INC. USA). 40 μl of the serum and 1 μl of 5 M luminol (5-amino-2, 3, -dihydro-1, 4-phthalazinedione) were combined and served as a probe. Levels of ROS were assessed by measuring the luminol-dependent chemiluminescence with the luminometer (BioTek Instruments, USA) in the integrated mode for 10 seconds. The results were expressed as relative light unit/ml (RLU/ml). Serum 8-OHdG was measured using a rat 8-Hydroxydeoxyguanosine ELISA Assay Kit (Cayman Chemical, USA) according to the instructions from the manufacturer. The concentrations of MDA in the serum and lens were determined using a lipid peroxidation assay kit (Jiancheng Bioengineering Institute, China), according to the instructions from the manufacturer. The total and soluble proteins of the lens were measured using the method of Lowry and by using a protein assay kit (Sigma) according to the manufacturer’s instructions. The amount of oxidized protein containing carbonyl groups was measured using an OxyBlot™ Protein Oxidation Detection Kit (Millipore, USA). Briefly, 20 μg of protein from the SDS extract were reacted with 1 × dinitrophenylhydrazine (DNPH) for 15–30 min, followed by neutralization with a solution containing glycerol and β-mercaptoethanol. The samples were electrophoresed on a 10% Tris–glycine gel, transferred, and blocked with 5% fat-free dry milk. The blot was incubated overnight with a rabbit anti-DNPH antibody (1:150) at 4 °C, followed by incubation in goat anti-rabbit (1:300) for 1 hour at room temperature. Bands were visualized using an ECL kit. All experimental protocol were approved by State Committee of Science and Technology of the People’s Republic of China, including any relevant details.

### Histology and immunohistochemistry

The paraffin-embedded sections (5-μm) of the lens were stained with hematoxylin and eosin (H&E) or Masson respectively and examined under a light microscope. The expressions of Nrf2 in the lens were characterized by immunohistochemistry using rabbit anti-rat Nrf2 antibody (1:100, Abcam, USA), biotinylated goat anti-rabbit IgG and the ABC staining kit (Golden Bridge Biotechnology, China). The images were performed in a single session using an Olympus microscope (Olympus BX71).

### Western blot analysis

The lens cytoplasm and nuclear protein extracts were separated by 10% sodium dodecyl sulfate polyacrylamide gel electrophoresis (SDS-PAGE) and then transferred onto PVDF membranes. The membranes were incubated with rabbit antibodies against Nrf2 (1:1000, Abcam, USA), TRX (1:1000, Abcam, USA), Catalase (1:2000, Abcam, USA), 4-HNE (1:1000, Abcam, USA), β-actins (1:2000, Abcam, USA) and Histone H2A (1:200, Santa Cruz, USA) respectively. The membrane was incubated with secondary antibody (horseradish peroxides-conjugated anti-rabbit IgG (1:5000, Habersham Biosciences, USA). The signal was detected using an ECL kit and the signals were measured using Gel-Pro analyzing software.

### Statistical analyses

SPSS13.0 software was used to analyze the data. All data were expressed as mean ± SEM and analyzed by one-way ANOVA. A p value less than 0.05 was considered statistically significant.

## Additional Information

**How to cite this article**: Wang, F. *et al.* DL-3-n-butylphthalide delays the onset and progression of diabetic cataract by inhibiting oxidative stress in rat diabetic model. *Sci. Rep.* **6**, 19396; doi: 10.1038/srep19396 (2016).

## Supplementary Material

Supplementary Information
