# Supplementary material for: DL-3-n-butylphthalide delays the onset and progression of diabetic cataract by inhibiting oxidative stress in rat diabetic model
Source: Sci Rep. 2016 Jan 13;6:19396. doi: 10.1038/srep19396 (PMC4725374; doi:10.1038/srep19396)
Supplement: Supplementary Information [file srep19396-s1.doc]

**Supplementary Information Title page:**

**Title:** DL-3-n-butylphthalide delays the onset and progression of diabetic cataract by inhibiting oxidative stress in rat diabetic model

**Author list:**

1. Fuxu Wang, Department of Hematology, the Second Hospital of Hebei Medical University, 215 Western Heping Road, Shijiazhuang 050000, China
2. Jia Ma, Department of Ophthalmology, the Second Hospital of Hebei Medical University, 215 Western Heping Road, Shijiazhuang 050000, China
3. Fei Han, Department of Digestology, the Second Hospital of Hebei Medical University, 215 Western Heping Road, Shijiazhuang 050000, China
4. Xiujin Guo, Department of Ophthalmology, the Second Hospital of Hebei Medical University, 215 Western Heping Road, Shijiazhuang 050000, Chin
5. Li Meng, Laboratorical center for Electron Microscopy, Hebei Medical University, 361 Eastern Zhongshan Road, Shijiazhuang 050017, China
6. Yufeng Sun, Department of Digestology, the Second Hospital of Hebei Medical University, 215 Western Heping Road, Shijiazhuang 050000, China
7. Cheng Jin, Department of Histology and Embryology, Hebei Medical University, 361 Eastern Zhongshan Road, Shijiazhuang 050017, China
8. Huijun Duan, Department of Pathology, Hebei Medical University, 361 Eastern Zhongshan Road, Shijiazhuang 050017, China
9. Hang Li* ,Department of Histology and Embryology, Hebei Medical University, 361 Eastern Zhongshan Road, Shijiazhuang 050017, China

Department of Pathology, Hebei Medical University, 361 Eastern Zhongshan Road, Shijiazhuang 050017, China

1. Ying Peng*,State Key Laboratory of Bioactive Substances and Functions of Natural Medicines, Institute of Materia Medica, Chinese Academy of Medical Sciences & Peking Union Medical College, Beijing 100050, China

***Corresponding author:**

1. **Hang Li**, M.D. Professor of Histology and Embryology

Department of Histology and Embryology, Hebei Medical University, 361 East Zhongshan Road, Shijiazhuang 050017, China.

Tel: 86-311-86266082 Fax: 86-311-86043026 E-mail :575601521@qq.com

2. **Ying Peng**, Ph.D. Professor of Pharmacology

Pharmacology Department, Institute of Materia Medica, Chinese Academy of Medical Sciences & Peking Union Medical College.No.1, Xiannongtan Street, Xuanwu District, Beijing 100050, China.

Tel: +86-10-63165173 Fax: +86-10-63017757 E-mail: [ypeng@imm.ac.cn](mailto:ypeng@imm.ac.cn)

**Figures**

**Figure.1**

**
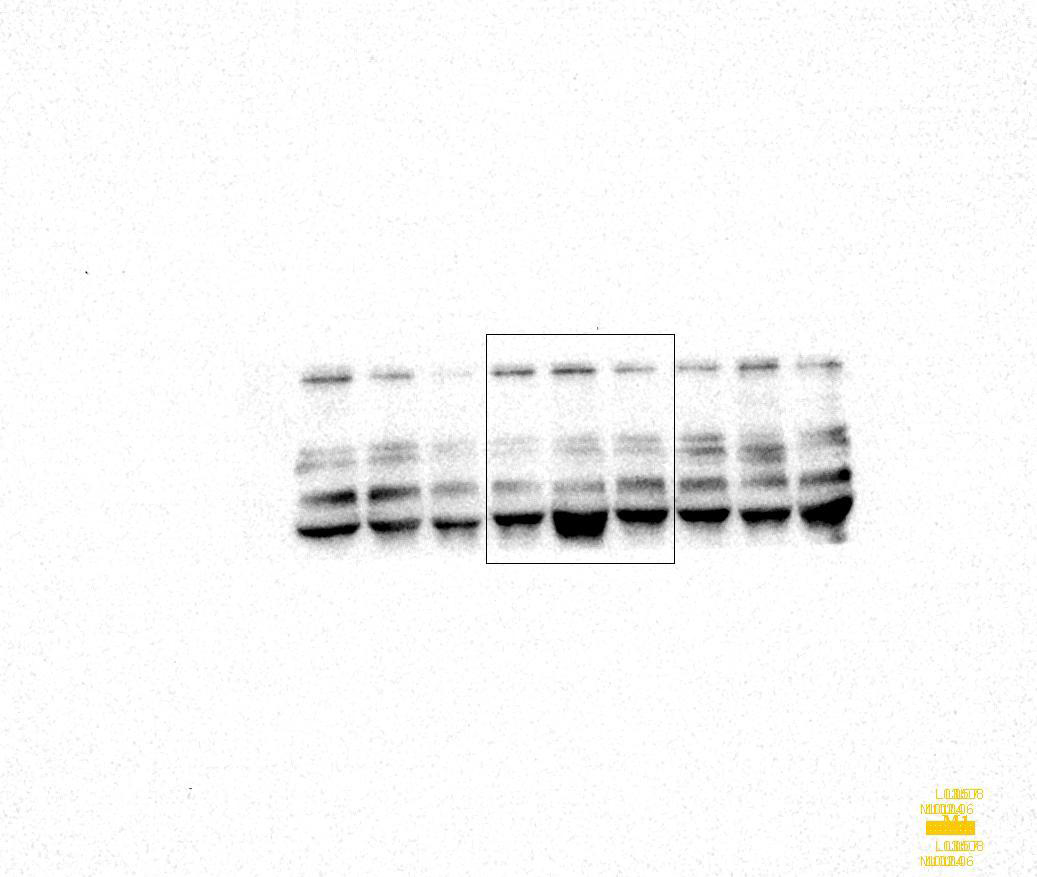
**

**Figure.1.** Expressions of lenses DNP by Western blot assays. Full-length blots of figure 3(a)


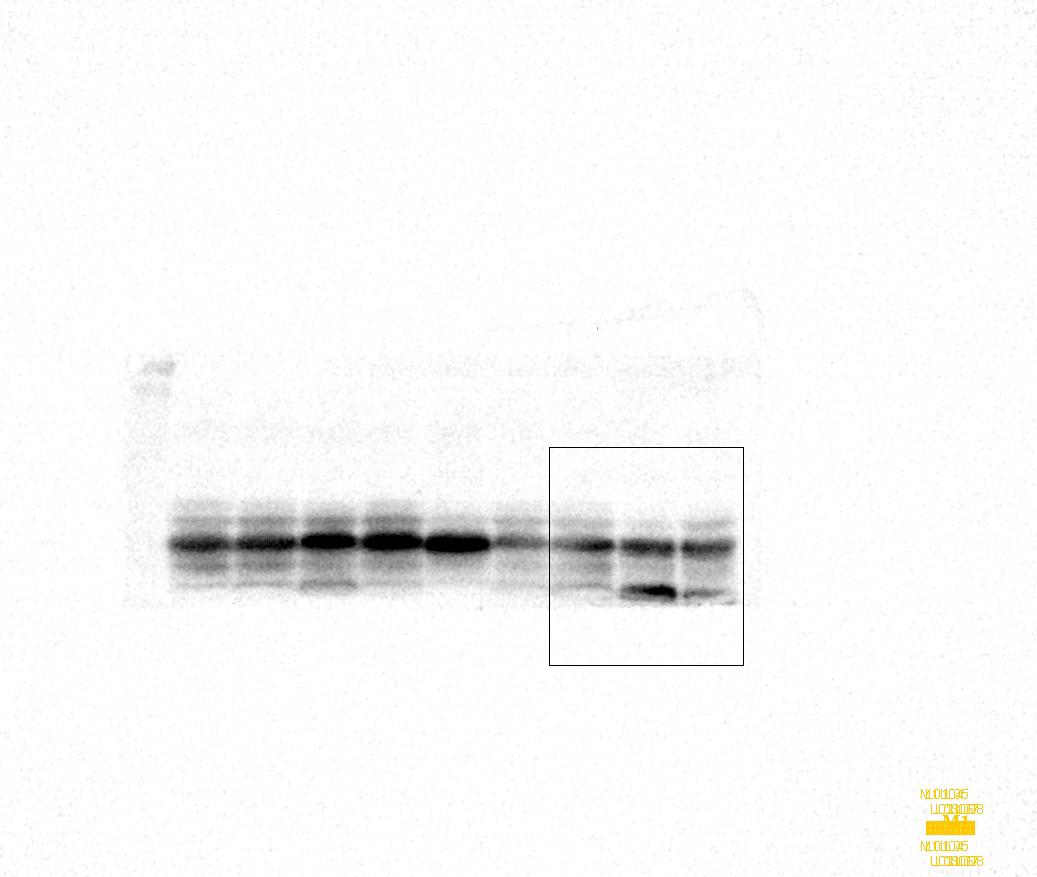
**Figure.2**

**Figure.2.** Expressions of lenses 4-HNE by Western blot assays. Full-length blots of figure 3(b)


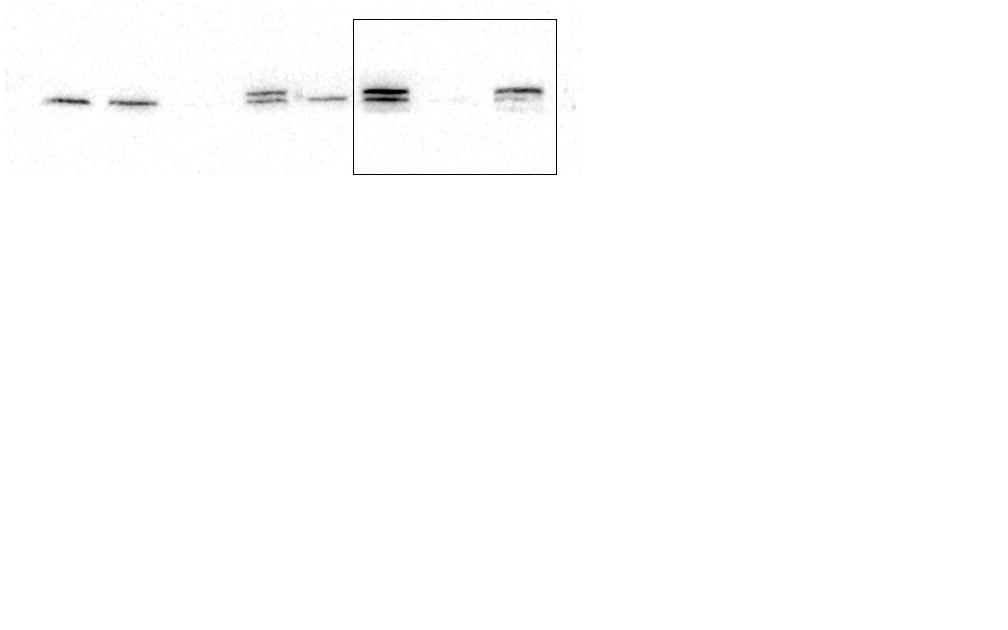
**Figure.3**

**Figure.3.** Expressions of lenses nuclear Nrf2 by Western blot assays. Full-length blots of figure 4(a).

**Figure.4**

**
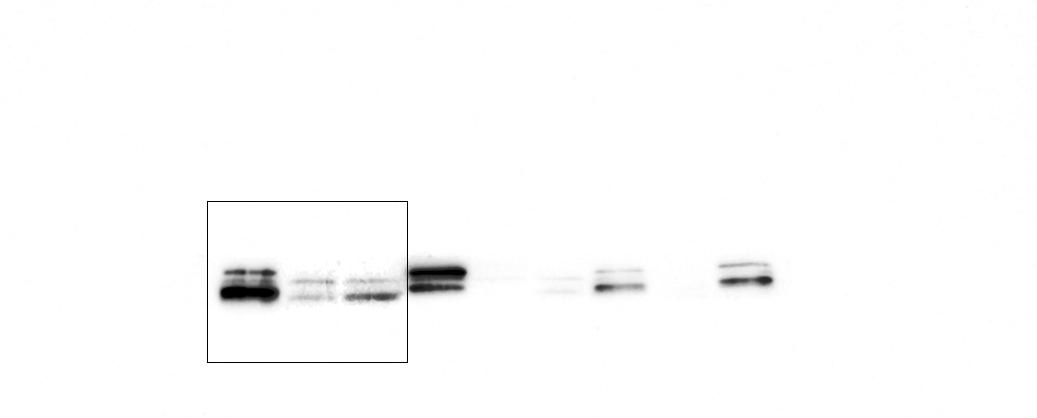
**

**Figure.4.** Expressions of lenses Nrf2 by Western blot assays. Full-length blots of figure 4(b).

**Figure.5**

**
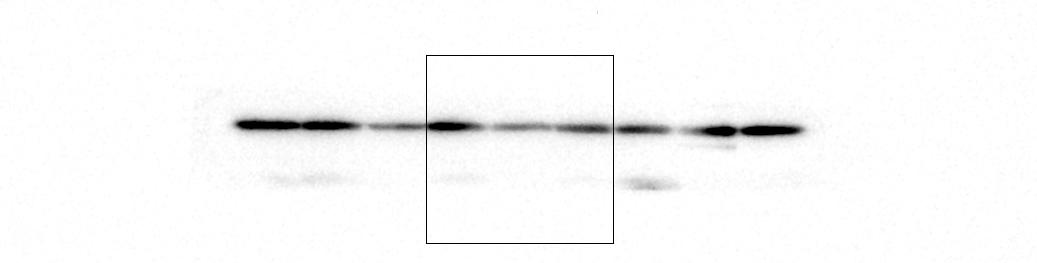
**

**Figure.5.** Expressions of lenses TRX by Western blot assays. Full-length blots of figure 4(d).

**Figure.6**

**
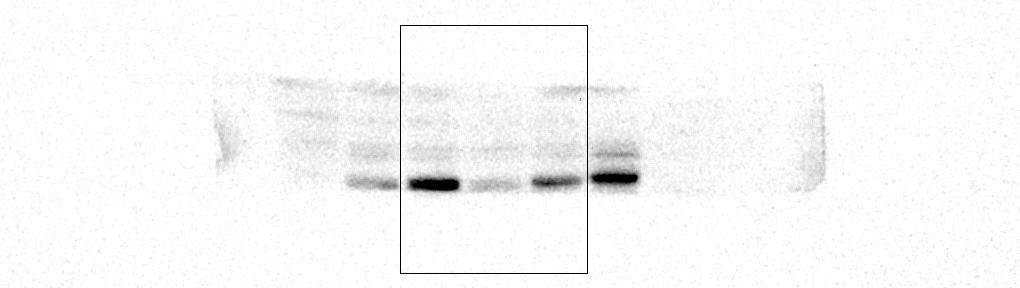
**

**Figure.6.** Expressions of lenses Catalase by Western blot assays. Full-length blots of figure 4(e).
